# Mechanisms of group B *Streptococcus*-mediated preterm birth: lessons learnt from animal models

**DOI:** 10.1530/RAF-21-0105

**Published:** 2022-06-07

**Authors:** Noble K Kurian, Deepak Modi

**Affiliations:** 1 Department of Microbiology, Atmiya University, Rajkot, Gujarat, India; 2 Molecular and Cellular Biology Laboratory, ICMR-National Institute for Research in Reproductive Health and Child Health (NIRRCH), Indian Council of Medical Research (ICMR), Mumbai, India

**Keywords:** *Streptococcus agalactiae*, pathogenesis, infection, membrane rupture, animal model, pregnancy

## Abstract

Group B *Streptococcus* (GBS) is an opportunistic pathogenic bacterium which upon colonization in the female reproductive tract can cause preterm births, fetal injury, and demise. Several determinants for GBS pathogenesis have been explored so far through the studies using animal models ranging from mice to non-human primates. The results from these experimental data have identified outer membrane vesicles, β-hemolysin, hyaluronidase, and Cas9 of GBS as major virulence factors leading to preterm births. Most of these factors drive inflammation through activation of NLRP3 and elevated production of IL1-β. However, the absence of one of the factors from the pathogen reduces but does not completely abolish the pathogenesis of GBS suggesting the involvement of more than one factor in causing preterm birth. This makes further exploration of other virulence factors of GBS pathogenesis important in gaining an insight into the mechanistic basis of GBS-mediated preterm births.

**Lay summary:**

Group B *Streptococcus* (GBS) is a pathogenic bacteria whose infection in the reproductive tract during pregnancy can cause premature delivery. This bacterial infection is one of the major causes of death of mother and baby during pregnancy, and the bacteria is prevalent in all parts of the world. This makes the research on GBS so important and many of the mechanisms behind GBS infection during pregnancy still remain unexplored. In this review, we have outlined how various animal models contributed in finding the mechanism of GBS pathogenesis. The review also focuses on compiling various virulence factors which makes GBS pathogenic in the vulnerable. Understanding the mechanisms of infection by GBS will be crucial in developing drugs and vaccines to protect against the harmful effects of the bacteria.

## Introduction

Preterm birth is defined as the delivery of the baby before 37 weeks of gestation. Worldwide 8–11% of all pregnancies result in preterm birth with some variation based on geographical locations and socioeconomic conditions (Shabayek & Spellerberg 2018, Walani 2020). Preterm birth is one of the leading causes of neonatal morbidity and mortality and is responsible for 75–80% of all neonatal deaths. Preterm birth can be classified into spontaneous and iatrogenic causes. Spontaneous preterm birth occurs due to regular uterine contractions or rupture of membranes prior to 37 weeks of gestation (Tucker & McGuire 2004), while iatrogenic preterm birth occurs due to provider-induced labor or cesarean section in the absence of spontaneous labor or rupture of membranes before 37 weeks of gestation (Chen *et al.* 2021). Spontaneous preterm births account for 65–70% of all preterm births and provider-initiated labor accounts for almost 30–35% of cases (Chen *et al.* 2021). In this manuscript, we will be discussing the role of infections in the occurrence of spontaneous preterm births.

While there are many causes of spontaneous preterm births, it is estimated that more than 40% of these occur as a result of intrauterine infections. In individual cases, it is difficult to assess whether the infection is the sole contributor to preterm delivery. However, several pieces of evidence ascertain that the infection and its resultant inflammation is the primary cause in a substantial proportion of preterm births. The evidence includes the presence of pathogenic microbes in the vaginal tract and elevated levels of inflammatory cytokines in the amniotic fluid of infants born preterm (Gervasi *et al.* 2012). In experimental models, intrauterine administration of the microbe has been shown to induce preterm birth (Elovitz & Mrinalini 2004). In addition, systemic or reproductive tract-specific maternal infections or subclinical intrauterine infection also contribute to preterm birth (Agrawal & Hirsch 2012). Using culture-independent methods and molecular phylogenetic approaches, distinct differences in the vaginal microbiota have been observed in women who delivered preterm as compared to those delivered at term (Romero *et al.* 2014). Recently, it was reported that changes in healthy populations of lactobacilli in the vagina to a mixed-species microbiota predominated by *Gardnerella vaginalis*, *Atopobium vaginae,* and *Prevotella* sp. are associated with preterm births (Crosby *et al.* 2018, Kumar *et al.* 2021). These observations are tantalizing evidence pointing toward a causal relationship between bacterial colonization and preterm births.

While the information on general microbial health in the lower reproductive tract of women with preterm birth is emerging from different parts of the world (McGregor *et al.* 1995, MacIntyre & Bennett 2021), several studies using classical culture-based methods have identified the presence of several pathogenic bacterial species in women who delivered preterm. *Lactobacillus crispatus, Lactobacillus iners, Lactobacillus gasseri, Lachnospiraceae BVAB1, Gardnerella vaginalis,* group B *Streptococcus*(GBS), etc. are consistently reported in clinical studies in various populations (Fettweis *et al*. 2019). Among these, the gram-positive GBS is the most extensively studied microbe in association with preterm birth. In the present communication, we present an overview of the causal relationship between GBS and preterm birth. Rather than being comprehensive, we will highlight the virulence factors identified so far and the mechanisms by which they cause preterm births.

## Group B *Streptococcus* and preterm births

Group B *Streptococcus* (*Streptococcus agalactiae*) is a facultative gram-positive β-hemolytic bacteria mainly associated with respiratory, genital, and gastrointestinal organs. It is an opportunistic pathogen of the female reproductive tract whose ascending infection in pregnancy is associated with adverse outcomes mainly preterm births (Vornhagen *et al.* 2018). Further, vaginal colonization of GBS has a direct correlation with vertical transmission of the pathogen (McDonald *et al.* 1989) and resultant invasive infections in the newborn leading to serious implications like pneumonia and meningitis in the newborn (Heath 2016). The associations between maternal GBS colonization and preterm birth were observed in multiple cross-sectional and case–control studies when cultures were performed at delivery (Ashary *et al.* 2022, Berikopoulou *et al.* 2021, Yaseen *et al.* 2021). However, this association was not observed in longitudinal cohorts where cultures were performed earlier in pregnancy (Bianchi-Jassir *et al.* 2017). These observations imply that GBS colonization in the third trimester is associated with preterm births.

A great variation on the prevalence of GBS has been observed in different geographical locations globally. A meta-analysis regarding the recto-vaginal colonization of GBS in 85 countries revealed that 35% of Caribean, North America, and Europe and 25% of South Africa were the regions where the most prevalent colonization was observed with a global average of approximately 20–25% being affected from GBS infections. In India, the carriage rate for GBS is estimated to be 7.4% (Ashary *et al.* 2022). The main factors responsible for GBS recto-vaginal colonization include biological factors like premature rupture of membranes, presence of GBS in the intestinal tract, and mother with age over 40. Numerous other factors including ethnicity, hygiene, illiteracy, obesity, etc. are also a reason for GBS colonization (Patras & Nizet 2018). The burden for colonization is very high as GBS is found to be the leading cause of preterm birth and stillbirth globally. A conservative analysis of international data for a single year revealed that about 3.5 million cases of preterm birth were associated with GBS infection. Another systematic review reported that 0.38 among 1000 pregnant women had invasive GBS infections which could even lead to maternal death. So GBS infections are considered to be so problematic for both mother and fetus (Brokaw *et al.* 2021).

## GBS infection models

To understand if GBS colonization is a cause of preterm birth or is merely an association, experimental studies in animal models are required. Since the 1970s, many GBS infection models have been developed (Larsen *et al.* 1978, Ancona & Ferrieri 1979, Cox 1982, Harrell *et al.* 2017). These include mice (Cox 1982), rats (Ancona & Ferrieri 1979), and primates like marmosets and rhesus monkeys (Rubens *et al.* 1991). Two major considerations need to be highlighted while interpreting the data from animal models: first, the strain of the bacterial species used and second, the animal system used. From the evidence gathered so far, in very few studies, clinical isolates have been used (Whidbey *et al.* 2015); most studies have only used the lab-adapted strains of GBS. Also, the lab-adapted GBS strains used in the studies till now are highly diverse. GBS strains belonging to serotypes I–V have been used of which type III is the one most extensively utilized (Ancona & Ferrieri 1979, Cox 1982, Patras *et al.* 2015, Whidbey *et al.* 2015, Vornhagen *et al.* 2016, Andrade *et al.* 2018). Mostly in the case of mice, it was found that the vaginal colonization efficiency of type V is excellent compared to type III and other strains (Patras *et al.* 2015).

The ability of GBS to persist in the mouse vaginal tract varies among the serotypes infected. The GBS strain, CJB111 (serotype V), persisted beyond several weeks in >50% of mice while GBS strains A909 (serotype Ia) and COH1 (serotype III) persisted for about 1–2 weeks. This effect is perhaps not due to differential immune responses. GBS colonization in the vaginal tissues resulted in significantly higher levels of keratinocyte-derived chemokine, IL-6, IL-1β, and macrophage inflammatory protein-2 (MIP-2), but no increased production of IL-23 was observed. However, no strain-specific differences in cytokine profiles were noted. However, when bacterial loads were quantified from these same mice, more numbers of CJB111 colony-forming units were recovered than the other strains (Patras *et al.* 2015). Thus, the cause of such differential ability to colonize needs to be identified. Nevertheless, these results imply that the GBS strains may use the same pathways to cause inflammation in the vaginal epithelium, whether this is true for other tissues in the feto–maternal interface needs to be determined.

Several mouse strains are utilized in studying GBS infection. Among these, Swiss Webster, CD-1, and C57BL/6J strains are mostly used in infection studies (Table 1). Patras and Doran (2016) developed a model which promotes prolonged persistence of GBS in rodent vagina. They demonstrated that administration of exogenous estrogen promoted sustained colonization of GBS A909 (American Type Culture Collection, ATCC #BAA-1138) strain persistence in CD-1 mice. The colonization was observed in almost 90% 2 weeks post-inoculation. Further, the CJB111 GBS strain persisted in the majority of CD-1 mice even beyond a month (Patras & Doran 2016). This persistent infection model will open new avenues in studying the long-term sequel of GBS colonization. It has been shown that intrauterine administration of GBS induces preterm labor in CD-1 and C57BL6/J mice strains (Whidbey *et al.* 2015). Of these two, the C57BL6/J strain is the most widely used model in GBS and preterm studies, where the administration of WT of GBS (Randis *et al.* 2014) or its heat-killed form (Equils *et al.* 2009) or even membrane vesicles (Surve *et al.* 2016) can cause preterm births. In all these studies, vaginal instillation of the microbe is a commonly followed procedure. Ancona and Ferrieri (1979) developed an albino rat model for investigating the mechanism of mother to fetal transfer of GBS as well its dynamics in vaginal colonization. Unlike the human vaginal mucosa, the superficial layers of the murine vaginal epithelium are highly keratinized, and therefore, the specific interactions underlying bacterial adherence may differ. Differences in vaginal pH, hormonal cycling, and the composition of the local microbiota must also be considered.

**Table 1:** Animal models used in studying GBS infections.

Animal model	GBS strains	Infection site	Infection time	Outcome	References
Mice					
Swiss-Webster	Type I a (SS-615), type I1 (SS-619), type 111 (DS-2434-80),	Vaginal	Three days before pregnancy	Colonization without disease	Cox 1982
C57BL/6J	Heat-killed GBS	Intrauterine, intraperitoneal	Embryonic day 14.5	Preterm-delivery	Equils *et al.* 2009
C57BL6/J	Type I b (H36B)		8-week-old female mice	NLRP3 inflammasome plays a crucial role in the control of *in vivo* GBS growth	Costa *et al.* 2012
C57BL6/J	Type V (NCTC 10/84)	Vaginal	8–12-week-old female mice	Preterm delivery/intrauterine fetal demise	Randis *et al.* 2014
C57BL6/J	Type I a (A909), Type III (COH1)	Intrauterine	Embryonic day 14.5	Fetal injury	Whidbey *et al.* 2015
CD-1	Type I a (A909)	Vaginal	Proestrus stage	Persistent vaginal colonization	Patras & Doran 2016
CD-1	Type V (CJB111), type I a (A909), type III (COH1)	Vaginal	Embryonic day 0	Differential host immune responses to different GBS strains	Patras *et al.* 2015
C57BL/6J	Type III (COH1)	Vaginal	Embryonic day 15	Ascending infection, *in utero* fetal demise	Vornhagen *et al.* 2016
C57BL6/J	Type I a (A909)	Intraamniotic	Embryonic day 14.5	Preterm birth and fetal injury	Surve *et al.* 2016
C57BL6/J	GB037	Vaginal	Embryonic day 13	Ascending vaginal infection	Kothary *et al.* 2017
BALB/c	Type III (BM110), attenuated isogenic mutant BM110ΔcylE	Vaginal	Day 17 and 18 of gestation	Enhanced mortality and higher bacterial load in lungs	Andrade *et al.* 2018
Rats					
Sprague-Dawley	Type la, type II, type III	Vaginal	Embryonic day 10.2	Mother–infant transmission	Ancona & Ferrieri 1979
Guinea pig					
*Cavia porcellus*	GBS mutants, hyperpigmented, GBS*covR*	Intrauterine	45 days of gestation	Increased dissemination into the amniotic fluid and fetal organs	Harrell *et al.* 2017
Non-human primates					
*Macaca mulatta*	Type I c, type III	Intraamniotic	130 days of gestation	Fetal meningitis and pneumonia	Larsen *et al.* 1978
*Macaca nemestrina*	Type III (COH-1)	Intraamniotic	140–145 days of gestation	Fetal lung injury	
*Macaca mulatta*	Type III	Intraamniotic	130 days of gestation	Increase in inflammatory proteins in amniotic fluid, fetal lung injury, meningitis	Gravett *et al.* 1994
*Macaca mulatta*	Type III	Intraamniotic/ choriodecidual	130 days of gestation	Preterm parturition	Gravett *et al.* 1996
*Macaca nemestrina*	Type III (COH-1)	Choriodecidua	118–125 days of gestation	Dysfunction of the cytokeratin network in amniotic epithelium	Vanderhoeven *et al.* 2014
*Macaca nemestrina*	Type III (COH-1)	Choriodecidua	118–125 days of gestation	Fetal lung injury	McAdams *et al.* 2015
*Macaca nemestrina*	GBS mutants, hyperpigmented, GBS*covR* nonpigmented GBS*covRcylE*	Choriodecidua	116–125 days of gestation	Preterm labor, fetal sepsis	Boldenow *et al.* 2016

The guinea pigs have been used in studying GBS infection (Table 1). Intrauterine inoculation of WT GBS in pregnant guinea pigs resulted in bacterial penetration into the placenta, amniotic fluid, and fetal organs (Harrell *et al.* 2017). Furthermore, hyperhemolysin-producing GBS strains showed a further increase in invasion into the amniotic fluid and fetal organs in guinea pigs. So, these animal models can be utilized as an effective tool in exploring the mechanism of action of various virulence factors of GBS in preterm births.

Among the various non-human primates, *Macaca nemestrina* and *Macaca mulatta* are the two most utilized non-primate models in studying GBS infections and preterm births. Studies on non-primates usually focused on exploring the effect of GBS instilled intraamniotically or choriodecidually in contrast to vaginal instillation in mice and hamster models (Table 1). Gravett *et al.* (1994) developed a chronically catheterized model of rhesus monkey (*Macaca mulatta*) and the infection was established by intraamniotic inoculation by GBS, type III strain. The model has an advantage that permits serial samplings of maternal/fetal blood and amniotic fluid on individual animals rather than the timed killing of animals.

In general, these models have been used to study the pathophysiology of intraamniotic effects of GBS such as inflammation and preterm births or effects on fetuses such as meningitis sepsis or lung injury (Table 1).

## GBS-mediated preterm births and premature rupture of the membranes (PROM) in experimental models

Considering preterm birth associated with GBS infections in context, it is important to understand how GBS induces preterm delivery. Whether GBS-mediated preterm births resemble the normal spontaneous parturition mechanism happening early or GBS activates other pathways. To understand this, Gravett *et al.* (1996) had analyzed the estrogen metabolism in GBS-infected dams to that of the control (without GBS infection) in rhesus monkeys. The results indicated that infection-associated parturition (either intraamniotic or choriodecidual) was characterized by abrupt increases in fetal DHEA, DHEA sulfate, androstenedione, progesterone, and cortisol, but there was no increase observed in maternal or fetal estrone or estradiol. This indicates that a normal spontaneous mode of parturition is not followed during GBS infection-associated preterm delivery.

Preterm premature rupture of membranes (PPROM) complicates about 30% of the preterm deliveries, of which, the majority of women (70%) with PPROM deliver within 24 h after membrane rupture. Inflammation in the fetal membranes (chorioamnion) and within the amniotic fluid is responsible for the rupture of membranes resulting in preterm birth. Infection-associated inflammation can lead to elevated cytokine levels, collagen remodeling, and membrane weakening leading to preterm delivery. Surve *et al.* (2016) reported that membrane vesicles of GBS contributed to collagen fragmentation and membrane stiffening in mouse choriodecidua. Along with collagen degradation, apoptosis of cells at choriodecidua was also observed. Both events contributed to membrane loosening leading to its rupture, PPROM, and preterm delivery.

In the non-human primate GBS infection model, similar deformities leading to PPROM and preterm births were observed (Vanderhoeven *et al.* 2014). GBS exposure to the choriodecidua resulted in the downregulation of genes mainly involved in maintaining the cytoskeleton like cytokeratins, collagen and collagen precursors, and intracellular matrix genes like laminins, desmocollin 2, and desmoplakin. This suggests that the early choriodecidual infection decreased cellular membrane integrity and tensile strength via dysfunction of cytokeratin networks, which may contribute to PPROM.

In GBS-infected pregnancies, there is a profound chance of fetal injury or death. Hemolytic GBS infection resulted in fetal demise and the bacteria was found to spread into fetal lungs and liver in mouse models (Randis *et al.* 2014). Similar sort of effects like fetal demise and infection in fetal organs were observed in higher models like guinea pigs and non-human primates (McAdams *et al.* 2015, Harrell *et al.* 2017).

## Identification of GBS virulence factors in experimental models

For GBS to cause preterm births, it needs to adhere to the vaginal epithelium, colonize there, ascend to the feto–maternal interface, and finally cause rupture of membranes. Several bacterial factors are identified to contribute to these steps.

## The two-component system of GBS

The transition of non-pathogenic vaginal colonizer to the pathogenic form of GBS is governed by many genetically encoded regulatory systems. One such system in GBS is the two-component system (TCS). The first component is the inner membrane-associated histidine kinase system and the second component is a cytoplasmic response regulator. GBS has 17–20 such TCS which plays an important role in its virulence. One of the well-characterized TCS in GBS is the control of virulence S (CovS) which is a sensor histidine kinase and its response regulator CovR. The CovS and CovR together regulate the expression of virulence genes like β-hemolysin, fibrinogen-binding protein (Fbs A, FbsB, and Fbs C), genes involved in iron uptake, antioxidant carotinoid pigments, etc. Other important GBS TCS include RgfA/C, HssRS, CiaR/H, LiaR/S, DltR/S, BgrR/S, FspS/R, NsrR/K, etc. coordinatively regulate virulence factors, stress response and AMP resistance (Poyart *et al.* 2001, Quach *et al.* 2009, Rozhdestvenskaya *et al.* 2010, Klinzing *et al.* 2013, Faralla *et al.* 2014, Khosa *et al.* 2016, Joubert *et al.* 2017) (Table 2). Of all these TCS systems, CovR/S is the most studied and proved *in vivo* to contribute to the vaginal colonization of GBS. Many other TCS systems which contribute to vaginal attachment as well as lantibiotic resistance were found to contribute in *in vitro* conditions and need to be confirmed *in vivo* (Patras & Nizet 2018).

**Table 2:** GBS two-component system (TCS).

GBS TCS	Function	Reference
CovR/S	Regulates the expression of virulence genes, helps in vaginal colonization	Patras & Nizet 2018
RgfA/C	Controls the expression of C5a peptidase which inactivates host complement-derived chemokines	Faralla *et al.* 2014
HssRS	Regulates heme metabolism which is important when colonizing blood-rich organs.	Joubert *et al.* 2017
CiaR/H	Provides resistance to host antimicrobial peptides	Quach *et al.* 2009
LiaR/S	Interacts with host AMP	Klinzing *et al.* 2013
DltR/S	Maintains the level of d-alanine in GBS cell wall which contributes to the AMP resistance	Poyart *et al.* 2001
BgrR/S	Controls the expression of β-antigen which contributes to GBS virulence	Rozhdestvenskaya *et al.* 2010
FspS/R	Regulates fructose metabolism which contributes to vaginal colonization	Faralla *et al.* 2014
NsrR/K	Regulates the genes involved in lantibiotic resistance which enable GBS to compete with the microbial flora	Khosa *et al.* 2016

Using various experimental animal models and GBS strains lacking certain genes (Hayes *et al*. 2020), the four major virulence factors that have emerged that contribute toward the pathogenesis of GBS-mediated preterm births include the GBS membrane vesicles (MVs), β hemolysin, hyaluronidase, and Cas9.

## Membrane vesicles of GBS and preterm births

An interesting phenomenon uncovered while exploration of the mechanism of GBS infection was the finding of MV of GBS (Surve *et al.* 2016, Kurian & Modi 2019, Mehanny *et al.* 2020, Armistead *et al.* 2021, McCutcheon *et al.* 2021). There exists experimental evidence to show that the MVs can induce preterm birth and fetal injury when administered prenatally (Surve *et al.* 2016) and aggravate morbidity and mortality of mice infected with GBS when administrated neonatally (Armistead *et al.* 2021). GBS MVs are nearly 50–300 nm in diameter and filled with virulence factors (Surve *et al.* 2016, McCutcheon *et al.* 2021). The GBS MVs can internalize in a range of cell lines including HeLa (Surve *et al.* 2016), human lung epithelial cell line (A549), human keratinocyte cell line (HaCaT), differentiated macrophage-like cells (dTHP-1), and murine dendritic DC2.4 (Mehanny *et al.* 2020). Intriguingly, these cells had good viability and there was negligible cytotoxicity even after 24-h incubation with MVs. Further, the non-immune cells have a higher ability to internalize and retain the GBS MVs as compared to immune cells (Mehanny *et al.* 2020). These results imply that GBS MVs can affect multiple cell types explaining the pleiotropic presentations of GBS infection (Lee *et al.* 2019). While such internalization is not cytotoxic, the MV cargo can alter intracellular gene expression and eventually alter homeostasis.

The GBS MVs are enriched with nucleic acids, certain lipids, and virulent factors including hyaluronate lyases, C5a peptidase, and sialidases (Surve *et al.* 2016, McCutcheon *et al.* 2021). There appears to be some strain-specific differences in the components of GBS MVs (Bohnsack *et al*. 1993, Chang *et al*. 2014) where only 62/643 MV proteins are common to six strains of GBS (McCutcheon *et al.* 2021) and these proteins can be the signature of the GBS MV proteome.

**Figure 1:**
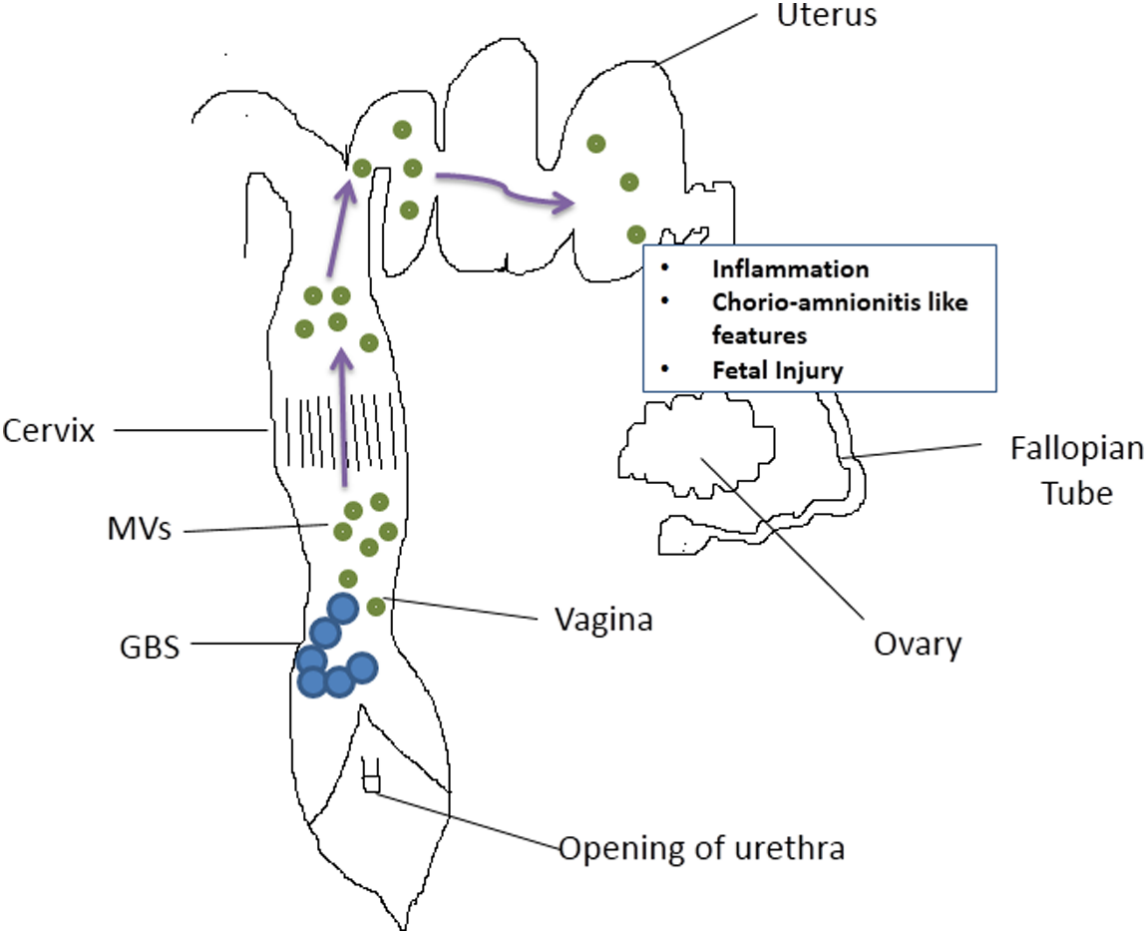
Mechanism of GBS infection mediated by membrane vesicles. GBS colonies in the vagina release membrane vesicles which move to the upper reproductive tract and can cause extensive collagen degradation and tissue destruction in fetal sacs resulting in fetal injury and preterm delivery.

While the anterograde movement of the bacteria was thought to be essential for the pathogenesis of GBS and cause preterm births, it was shown that fluorescently labeled MVs from GBS strain A909 when instilled in mouse vagina (C57BL6/J strain) could undergo anterograde movement (Surve *et al.* 2016). Furthermore, intra-amniotic injection of MVs to the fetal sacs resulted in extensive collagen degradation and tissue damage. Intraamniotic injections of MVs were sufficient to result in chorioamnionitis and an increase in the expression of inflammatory cytokines similar to those reported in women with preterm births (Surve *et al.* 2016). Further, MVs in the amniotic sac resulted in intrauterine fetal death and preterm delivery (Surve *et al.* 2016). Thus, the MVs produced by GBS were sufficient to mimic phenotypes of the infection without the physical presence of the microbe (Fig. 1). A recent study has shown that MVs from hyperhemolytic GBS strains were more pathogenic on neutrophils, T cells, and B cells compared with MVs from nonhemolytic GBS (Armistead *et al.* 2021) suggesting that a granadaene-mediated virulence of GBS is mediated via MVs.

## β-hemolysin as a pathogenic factor

To establish the colonization at the female genital tract, GBS must adhere to the vaginal epithelium successfully. GBS binds very efficiently to the epithelium in acidic vaginal pH (Shabayek & Spellerberg 2018). Several factors promote GBS binding to the vagina and subsequent ascension. The low-affinity interaction of GBS with the vaginal epithelium is mediated by its cell wall-associated lipoteichoic acid while high-affinity interactions are mediated by extracellular matrix proteins like fibronectin, laminin, and others which interact with host cell integrins (Doran & Nizet 2004). The hemolysin-producing GBS was shown to have an edge in colonizing the vagina (Edwards *et al.* 2016).

An important step in GBS pathogenesis is its anterograde transition from the vagina to the fetal sac. Based on animal studies, specifically the mice, the role of a few virulence factors involved in GBS ascension and infection had been established. Randis *et al.* (2014) for the first time demonstrated that β-hemolysin/cytolysin (βH/C) adversely affects pregnancy outcomes following maternal vaginal colonization in C57BL6/J mice. In this study, competition assays demonstrated a marked advantage to βH/C-expressing GBS during colonization. Intrauterine fetal demise and/or preterm birth were observed in 54% of pregnant mice colonized with WT GBS vs none with the strains deficient for βH/C. In another study with vaginal administration, six different hyperhemolytic covR strains of GBS resulted in inducing preterm birth compared to WT strain (Whidbey *et al.* 2015). Both these studies together underscore the key role of bacterial is β-hemolysin as a pathogenic factor for preterm births.

**Figure 2:**
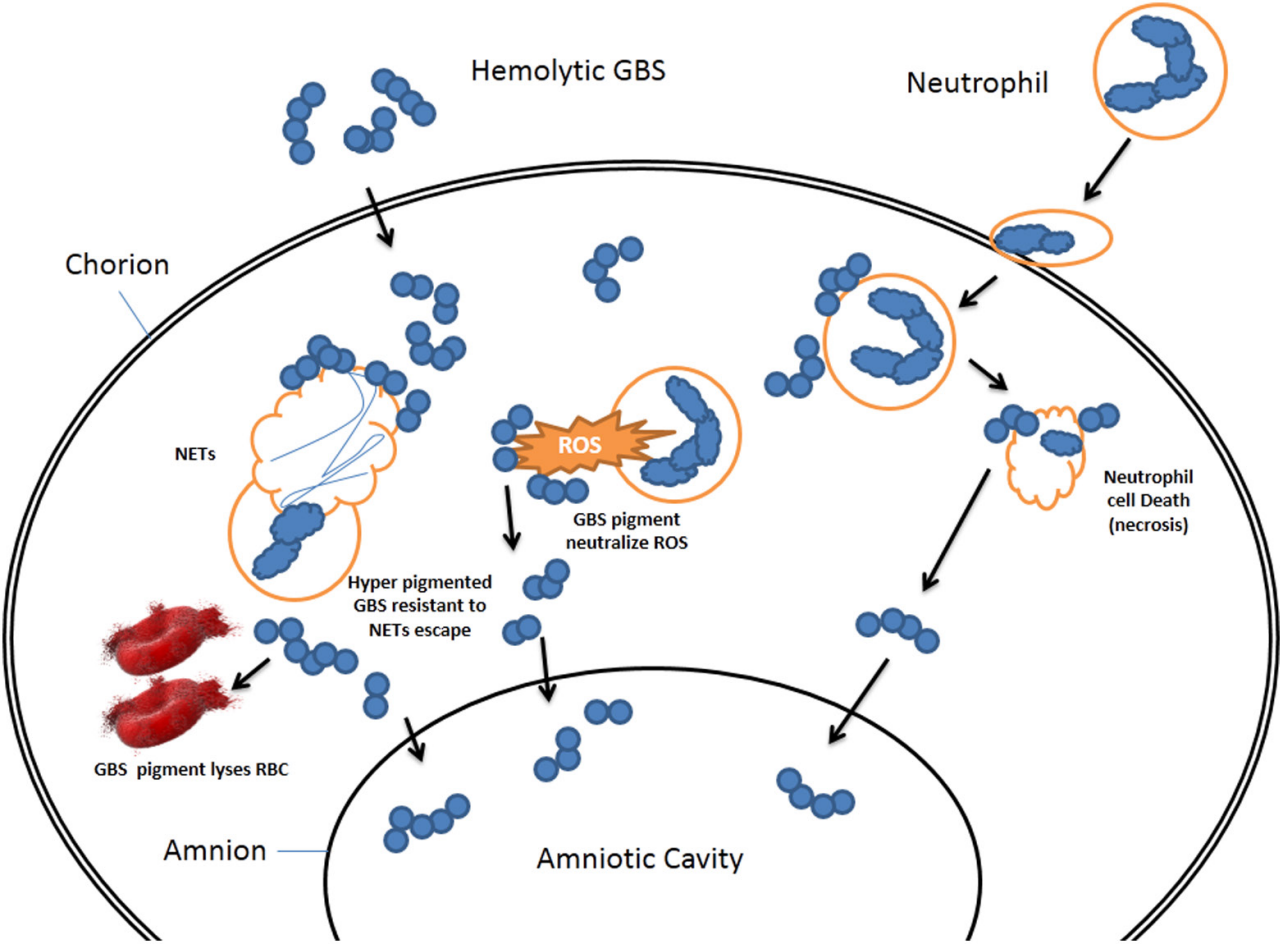
GBS induces NLRP3 inflammasome-dependent programmed cell death. GBS pigment hemolysin can activate NLRP3 inflammasome and thereby lead to cell death (pyroptosis or injury leading to fetal death).

Beyond preterm births, in the context of virulent factors, the effect of hemolysin and its mechanism has been well dissected. Robust inflammation at the feto-maternal interface is a key feature of GBS infections (Costa *et al.* 2012, Boldenow *et al.* 2016, Surve *et al.* 2016). The increase in the secretion of IL-1β and IL-18 observed in human macrophages treated with GBS pigment (β-hemolysin) suggests that the pigment can trigger activation of the inflammasome (Costa *et al.* 2012). Pregnant homozygous NLRP3 knockout mice (NLRP3KO) were utilized to determine whether the hemolysis and/or activation of the NLRP3 inflammasome is important for fetal injury and preterm birth caused by hyperhemolytic GBS strains (Brydges *et al.* 2009, Kovarova *et al.* 2012). Notably, preterm delivery was observed in 3/6 WT C57BL6 mice infected with ΔcovR and not in any other groups. Fetal death was significantly higher in NLRP3 knockout mice infected with ΔcovR compared to that infected with ΔcovRΔcylE indicates hemolytic/membrane-disrupting nature of the pigment (without NLRP3 inflammasome activation) is also likely to contribute to fetal injury. The results indicated that GBS infection-mediated fetal death is associated with the production of hemolytic pigment and the presence of NLRP3 inflammasome (Fig. 2). It was found that the production of hemolytic pigment contributes to GBS infection-associated fetal injury in both an NLRP3 inflammasome-dependent and NLRP3 inflammasome-independent manner (Whidbey *et al.* 2015). This further confirms the crucial role of GBS hemolysin in bacterial virulence in mouse models.

In non-human primates, the association of hemolysin with inflammation is widely explored. Infection of hyperpigmented hemolytic GBS in non-human primate model increased the levels of cytokines like IL-1β, tumor necrosis factor-α (TNF-α), IL-6, and IL-8 in amniotic fluid. The hemolytic GBS pigment was shown to induce neutrophil death by the lytic or necrotic manner is contrary to what was observed with macrophages (apoptosis/pyroptosis). Hemolytic GBS was found to induce the formation of neutrophil extracellular traps (NET) and the bacteria were found to be resistant to the NET in placental membranes *in vivo* (Boldenow *et al.* 2016).

**Figure 3:**
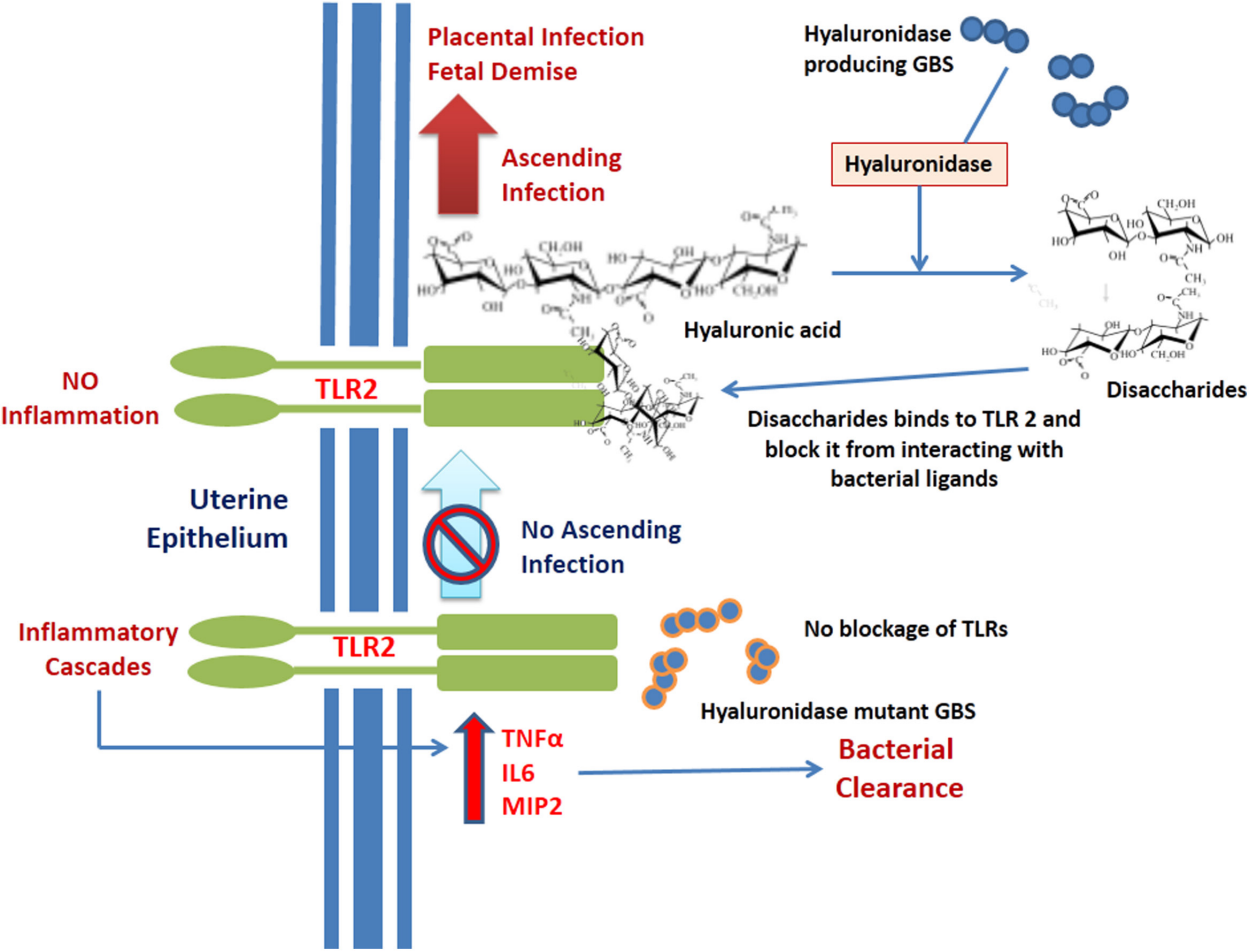
GBS pigment (hemolysin) and infection mechanisms. The GBS pigment lyse RBC as well as neutrophils and bypass the NETS as well as ROS to reach the amniotic cavity.

In summary, both in the mouse model and non-primate model, hemolysin-producing GBS is found to elicit inflammatory responses and resultant fetal injury, which confirm the devastating nature of the pigment molecule (Fig. 3).

## Hyaluronidases as a GBS pathogenic factor

After successful attachment, GBS needs to ascend to the placental membranes and amniotic fluid to reach the fetus to cause serious infection and damage. Several factors contribute to the travel of GBS from the vagina to the fetus. Among these, hyaluronidase or hyaluronate lyases, an exolytic enzyme, was found to contribute to GBS ascension.

**Figure 4:**
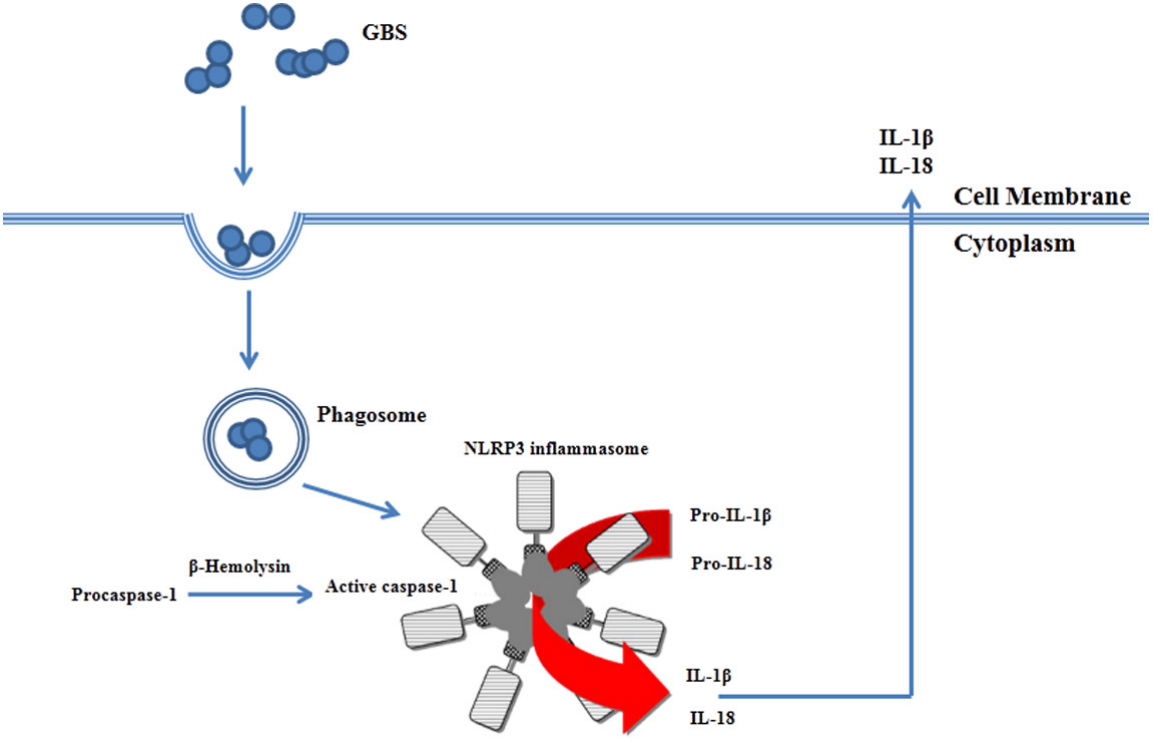
GBS hyaluronidase and its role in ascending Infection. Hyaluronidase produced by GBS can cleave the epithelial extracellular matrix component hyaluronic acid. The resulting product can block TLR2 which in turn leads to immunosuppression makes the ascending infection possible. But the non-hyaluronidase mutant GBS was found to be cleared by immune responses as they lack the enzyme.

The ascended bacteria need to break the maternal–fetal barrier so as to reach the fetus. Vornhagen *et al.* (2016) found that GBS hyaluronidases (HylB) degrade hyaluronic acid into disaccharide fragments which in turn bind to Toll-like receptors 2 and 4, thereby blocking the proinflammatory cascades against GBS ligands (Fig. 4). The proof that GBS hyaluronidases are key for ascending infections came from studies where C57BL/6J mice were vaginally inoculated with WT and HylB mutant GBS (GBSΔhylB) and the results revealed that HylB mutants shown less migration to the upper reproductive tract as compared to WT GBS strain (Vornhagen *et al.* 2016).

HylB cleaves the high-molecular-weight polymer hyaluronic acid and the resulting product blocks the TLR2 receptors involved in immune responses. This immune suppression mediated by HylB can help GBS to escape from immune responses, and this could result in devastating effects like preterm births and fetal injury (Henneke *et al.* 2008, Vornhagen *et al.* 2016). Despite the contribution of virulence factors like HylB in blocking immune responses, GBS normally elicits non-strain-specific immune responses in animal models tested. Studies in C57BL/6J mice demonstrated that HylB mutants of GBS increased the expression of inflammatory markers in uterine tissue compared to the WT GBS strains (Vornhagen *et al.* 2016). This denotes the immunosuppressive property of HylB. So GBS hyaluronate lyase can be considered a critical factor that promotes ascending infection by blocking immune responses in the uterine tissues, finally, resulting in preterm birth (Fig. 4).

## Endonuclease effector Cas9 as GBS virulence factor

Recent studies on C57BL/6 and CD-1 revealed that in type II GBS, endonuclease effector Cas9, which is a part of CRISPR/Cas locus, plays an important role in vaginal persistence and disease. Cas9 mutants of GBS had shown less persistence in the vaginal epithelium (Spencer *et al.* 2019). Also, differential expression of virulence factor genes is observed in Cas9 mutants (Spencer *et al.* 2019). This signifies that Cas9 can act as a regulatory factor in GBS which can influence the virulence of the pathogen. More knowledge regarding the non-canonical role of Cas9 in the regulation of pathogen colonization and disease will provide more insights into GBS pathogenesis in the future.

## Summary and conclusions

To date a few of the virulence determinants of GBS, namely MVs, β hemolysin, hyaluronidase, and Cas9 have been explored so far. The mechanisms by which these factors cause preterm births have been characterized to a reasonable extent. The study that has emerged so far indicates that most of these virulence factors activate inflammation at the feto–maternal interface. This inflammation in turn causes parturition-like changes causing preterm births. This inflammation can be caused by activating the NLRP3-mediated inflammasomes through various pathways including TLR activation. However, it must be noted that the absence of one of the factors does not always limit the bacteria to cause preterm births. This makes further exploration of virulence factors of GBS pathogenesis important.

The devastating nature of GBS infections gives an alarm that extensive screening for GBS is needed during pregnancy, which is lacking mainly in developing countries. Understanding more about GBS pathogenesis will help in developing effective vaccines and therapy against the pathogen.

## Declaration of interest

The authors declare that there is no conflict of interest that could be perceived as prejudicing the impartiality of the research reported.

## Funding

D M lab and is supported by grants from the Indian Council of Medical Research, Government of India. N K was a recipient of the Kerala State Council for Science, Technology & Environment Post-Doctoral Fellowship. The manuscript bears the NIRRH ID: REV/1168/11-2021.

## Author contribution statement

Both N K and D M conceived the idea and wrote the manuscript.
